# Time-Dependent Shifts in Intestinal Bacteriome, *Klebsiella* Colonization and Incidence of Antibiotic-Resistance Genes after Allogeneic Hematopoietic Stem Cell Transplantation

**DOI:** 10.3390/biomedicines12071566

**Published:** 2024-07-15

**Authors:** Oleg V. Goloshchapov, Alexey B. Chukhlovin, Dmitrii E. Polev, Yury A. Eismont, Dmitry S. Bug, Alexey V. Kusakin, Oleg V. Kosarev, Ruslana V. Klementeva, Vladimir V. Gostev, Vladimir A. Ageevets, Nikita P. Volkov, Anastasia S. Ipatova, Ivan S. Moiseev, Anna A. Spiridonova, Sergey V. Sidorenko, Alexander D. Kulagin

**Affiliations:** 1 R. Gorbacheva Memorial Research Institute of Pediatric Oncology, Hematology and Transplantation, Pavlov First State Medical University of St. Petersburg, L. Tolstoy St, 6-8, 197022 St. Petersburg, Russia; golocht@yandex.ru (O.V.G.); bug.dmitrii@yandex.ru (D.S.B.);; 2 Pasteur Institute of Epidemiology and Microbiology, Mira St, 14, 197101 St. Petersburg, Russia; 3 Pediatric Research and Clinical Center for Infectious Diseases, Prof. Popov St, 9, 197022 St. Petersburg, Russia; 4 Department of Informatics and Computer Technologies, St. Petersburg Mining University, 199106 St. Petersburg, Russia; 5 Department of Medical Microbiology, North-Western State Medical University Named after I.I. Mechnikov, Piskarevskij Ave, 47, 195067 St. Petersburg, Russia

**Keywords:** hematopoietic stem cell transplantation, allogeneic, gut microbiome, changes, time-dependent, DNA sequencing, NGS, *Bacillota*, *Enterobacteriaceae*, *Klebsiella pneumoniae* colonization, antibiotic resistance genes, CHROMagar technique, MDR, ESBL, carbapenem resistant, OXA-48, KPC, NDM

## Abstract

Dose-intensive cytostatic therapy and antibiotic treatment in allogeneic hematopoietic stem cell transplantation (allo-HSCT) cause severe abnormalities in a composition of gut microbiota as well as the emergence of antibiotic resistance. The data on the longitudinal recovery of major bacterial phyla and the expansion of genes associated with antibiotic resistance are limited. We collected regular stool samples during the first year after allo-HSCT from 12 adult patients with oncohematological disorders after allo-HSCT and performed 16SrRNA sequencing, multiplex PCR, conventional bacteriology and CHROMagar testing. We observed a decline in Shannon microbiota diversity index as early as day 0 of allo-HSCT (*p* = 0.034) before any administration of antibiotics, which persisted up to 1 year after transplantation, when the Shannon index returned to pre-transplant levels (*p* = 0.91). The study confirmed the previously shown decline in Bacillota (Firmicutes) genera and the expansion of *E. coli*/*Shigella*, *Klebsiella* and *Enterococci*. The recovery of *Firmicutes* was slower than that of other phyla and occurred only a year post-transplant. A positive correlation was observed between the expansion of *E. coli*/*Shigella* genera and *bla*_KPC_, *bla*_CTX-M-1_ and *bla*_TEM_ (*p* < 0.001), *Klebsiella* spp. and *bla*_OXA-48_-like, *bla*_NDM_, *bla*_CTX-M-1_, *bla*_TEM_, and *bla*_SHV_ (*p* < 0.001), *Pseudomonas* spp. and *bla*_NDM_ (*p* = 0.002), *Enterococcus* spp. and *bla*_OXA-48_-like, *bla*_NDM_, *bla*_CTX-M-1_, *bla*_SHV_ (*p* < 0.01). The correlation was observed between the expansion of Enterobacterales and and carbapenemase-positive CHROMagar samples (*p* < 0.001). Samples positive for carbapenem-resitant bacteria were at their maximum levels on day +30, and were gradually diminishing one year after allo-HSCT. From day +30 to +60, all isolated *K. pneumoniae* strains in fecal samples proved to be resistant to the main antibiotic groups (carbapenems, aminoglycosides, fluoroquinolones, third-generation cephalosporins). One year after HSCT, we documented the spontaneous decolonization of *K. pneumoniae*. The sensitivity of molecular biology techniques in the search for total and antibiotic-resistant *Klebsiella* seems to be superior to common bacteriological cultures. Future studies should be focused on searching for novel approaches to the efficient reconstitution and/or maintenance of strictly anaerobic microbiota in oncological patients.

## 1. Introduction

Allogeneic hematopoietic stem cell transplantation (allo-HSCT) is a standard method of therapy for high-risk hematological malignancies. This therapeutic procedure is, however, associated with the dramatic exhaustion of myeloid and lymphoid cell lineages thus causing the sufficient suppression of cellular immunity in the first months after HSCT. Therefore, the dissemination of commensal infectious pathogens is common in these patients, causing severe post-transplant infections, prolonged hospitalization terms and increased mortality [1]. Preventive anti-infectious therapy, either empiric or targeted, is routinely administered in the first month after HSCT; however, this leads to the disruption of the normal intestinal microbial community (microbiome) colonizing mucous surfaces. In clinical settings, the drug-induced damage to microbiota is diagnosed by routine bacteriological tests. Moreover, the early and late colonization of the oral cavity and other sites with antibiotic-resistant bacterial strains of *Klebsiella* was observed 1–2 months after HSCT [2].

A detailed characterization of the gut microbiome with common bacteriological methods is not possible, since most intestinal bacteria do not grow in standard aerobic cultures. This methodological gap is resolved by specific DNA diagnostic approaches, e.g., over the last decade, the biodiversity of the entire gut microbiota was studied by means of multiplex PCR and, more recently, using the next-generation sequencing (NGS) of the 16S rRNA gene, a well-mapped segment of the bacterial genome. The DNA diagnostics allow for the detection of fastidious and non-culturable anaerobic bacteria, which comprise the bulk of the intestinal microbiota. Serial molecular biology assays of real-world microbiota samples based on 16S rRNA gene sequencing allowed for the evaluation of bacteriome diversity (up to 1000 microbial species) of different mucous surfaces, stool specimens, oral fluids, bronchoalveolar secretions, urine, etc. The clinical significance of the DNA diagnostics of microbiota is still being researched due to the absence of appropriate standards for the biomaterial processing, bacterial DNA isolation and bioinformatic evaluation of NGS databases.

Several studies on stool samples after HSCT have shown a reduction in the biodiversity of intestinal microbiota following intensive cytostatic and anti-infectious therapy followed by HSCT. The authors observed the selective exhaustion of certain bacterial genera that are important for metabolic homeostasis. The biodiversity and relative abundance of these anaerobic clostridia, e.g., *Faecalibacterium*, *Roseburia, Ruminococcus, Blautia,* was sufficiently reduced soon after HSCT [3]. This exhaustion of the intestinal microbiota may be related to severe immune complications causing graft-versus-host disease (GvHD) and early transplant-associated mortality. Alternatively, the higher abundance of some other bacteria (e.g., *Eubacterium limosum*) in the gut microbiota seems to be associated with a lower risk of leukemia relapse in the treated patients [4].

Studies of time-dependent changes in the bacterial spectrum after HSCT are mostly limited to intestinal microbiota. Appropriate studies in other sites of human body, e.g., the bronchoalveolar lavage, are mostly limited by routine microbiology data [5].

Hence, the aim of present study was to evaluate basic time-dependent changes in dominating phyla and genera in gut microbiota, the time course of *Klebsiella pneumoniae* colonization and some common antibiotic resistance genes (ARGs) of intestinal bacteria from the pre-transplant period to day +365 after allo-HSCT.

## 2. Materials and Methods

### 2.1. Ethical Statement

The biological samples from stool used for this medical examination were taken with the approval of the attending physician. The study was conducted in accordance with guidelines of the 1964 Declaration of Helsinki and its later amendments. All patients or their guardians signed a written informed consent form for a hematopoietic stem cell transplant and the subsequent medical procedures, as well as the potential usage of their clinical data for the purposes of clinical research. This study was approved by the Local Review Board of the Pavlov First State Medical University of St. Petersburg (ID number 214 of 17 December 2018).

### 2.2. Patients and Study Design

We observed a group of 12 adult patients with different oncohematological disorders who underwent allogeneic HSCT. Their median age was 33 years old (Table 1). The underlying hematological disorders were as follows: acute myeloid leukemia (AML, *n* = 5); acute lymphoblastic leukemia (ALL, *n* = 3); chronic myeloid leukemia (CML, *n* = 3); non-Hodgkin lymphoma (NHL, *n* = 1). Prior to HSCT, the patients were heavily pre-treated in the course of the induction and consolidation therapy. The conditioning regimen included fludarabine and busulfan in 11 of 12 cases. Detailed clinical characteristics of the HSCT procedures and post-transplant complications are presented in Table 1.

Oral mucositis grade was determined according to the WHO scale [6]. Acute graft-versus-host disease (aGVHD) was classified at a standard scale [7].

### 2.3. Laboratory Studies

#### 2.3.1. Bacteriology

The incubation of blood cultures and other normally sterile biological fluids was performed in automated blood analyzers BacT/ALERT 3D 120 (BioMerieux, Craponne, France), or Junona LABSTAR (SCENKER Biological Technology, Liaocheng, China) with a prolonged monitoring system. The microbial species were identified via mass spectrometry with VITEK MS equipment (BioMerieux, Craponne, France) based on MALDI-TOF technology.

Clinical laboratory cultures were formed and the isolation of bacteria from other biomaterials was achieved in differential culture media using classical bacteriological techniques. Bacterial isolates obtained from stool samples were identified by means of commercial biochemical test systems as well as with MALDI-TOF mass spectrometry. The sensitivity of clinical isolates to common antibiotics was determined by means of standard disk-diffusion test systems according to the EUCAST standards [8].

Screening for extended-spectrum β-lactamase (ESBL)-positive Gram-negative bacteria in rectal swab samples was performed by seeding on a chromogenic medium (CHROMagar ESBL). To detect carbapenem-resistant Gram-negative bacteria, CHROMagar KPC cultures were used. Vancomycin-resistant enterococci (VRE) were identified by CHROMagar VRE test system (CHROMagar, Saint-Denis, France). Moreover, the species identity of all bacteria isolated from chromogenic media was confirmed by MALDI-TOF mass spectrometry.

#### 2.3.2. Multiplex PCR

Along with the NGS analysis, the relative amounts of clinically sound intestinal bacteria were assessed by a multiplex PCR test system (Enteroflor, DNA Technology, Moscow, Russia), which detected 24 bacterial species and genera in stool samples: *Bacteroides* spp., *Enterobacterales*, *Pseudomonas* spp., *E. coli*, *F. prausnitzii*, *Akkermansia muciniphila*, *Lachnospiraceae*, *C. difficile*, *Streptococcus* spp., *Str. agalactiae*, *Staphylococcus* spp., *St. aureus*, *Bifidobacterium* spp., *Lactobacillaceae*, *Enterococcus* spp., etc. Moreover, a semi-quantitative assay of some common ARGs in these samples was performed with BakResista PCR test system (DNA Technology, Moscow, Russia), i.e., *oxa-40-like*, *oxa-48-like*, *oxa-23-like*, *oxa-51-like*, *imp*, *kpc*, *ges*, *ndm*, *vim* (carbapenemases), as well as *tem*, *ctx-M-1*, *shv* (beta-lactamases). In brief, DNA was extracted from the supernatants of homogenized stool samples after enzyme lysis and 1 h incubation followed by magnetic bead absorption and elution, and was then subject to PCR.

The data were expressed as log_10_ of gene copies per mL of sample.

Statistical inter-group comparisons were made according to the parametric and non-parametric criteria using Statistica 5.0 software (Dell, Round Rock, TX, USA). The confidence level of *p* ≤ 0.01 was taken as significant.

#### 2.3.3. NGS-Based Assessment of Gut Bacteriome

The stool samples were collected in all patients in a period ranging from pre-transplant terms and covering the entire post-transplant time period (up to 360 days). Fecal samples were collected in all patients at ten time points: before HSCT and on days 0, +10, +20, +30, +60, +90, +120, +150, +180, +365 after HSCT. We collected 8 to 11 stool samples from each patient at different time points without repeated sampling. However, DNA extraction was repeated in cases where inferior DNA quality was obtained for NGS. The raw data of NGS file reads are available upon request from the authors.

The samples were quickly frozen and stored at −40 °C until DNA isolation. To prepare the specimens for NGS, DNA from glass-homogenized specimens was purified by the column method using QIAamp DNA Microbiome Kit (Qiagen, Hilden, Germany). The amount of total DNA was determined via NanoDrop photometry. The samples of sufficient quality were prepared for NGS as recommended by the manufacturer (Illumina, San Diego, CA, USA).

The biological diversity and composition of fecal microbiota were evaluated by means of 16S rRNA gene sequencing (V3–V4 regions) using MiSeq platform (Illumina) with v3-600 MiSeq reagents kits (Illumina).

#### 2.3.4. Bioinformatics

A bioinformatic analysis of NGS database was performed by R software using DADA2 pocket [9]. This pipeline included the filtration and assembly of the paired-end reads, obtaining the amplicon sequence variants (ASV), the removal of chimeric sequences, as well as taxonomic annotation by means of the Silva 138 reference database [10]. Moreover, Phyloseq pockets were applied in order to format the obtained data [11] and used to perform in silico decontamination [12]. The levels of microbiota biodiversity were determined as Shannon and Simpson indexes.

The rarefaction curves showed a sufficient trend to reach a plateau for NGS reads at a moderate ASV (amplicon size variants). At higher ASV numbers (>300), the plateau was not reached, obviously, due to the high representation of rare microbial species in the samples (Supplementary Figure S1). Still, the rarefaction methodology is still under discussion [13].

## 3. Results

### 3.1. Posttransplant Changes in Intestinal Microbiota

#### 3.1.1. Phylum Level

**Figure 1:**
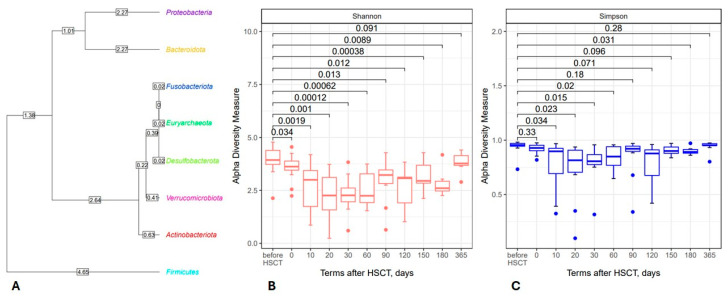
Left side: Phylogenetic tree of intestinal microbiota derived from the NGS database for 12 HSCT patients (**A**); most common genera in the general sample belong to *Firmicutes* (*Bacillota*). Right side: The box plots show changes in the Shannon (**B**) and Simpson (**C**) biodiversity indexes for the total fecal bacteriome at distinct time points. Abscissa; terms after HSCT; days. Ordinate; median biodiversity values and their ranges.

The results of 16S rRNA sequencing revealed eight major phyla of intestinal microbiota (Figure 1A). A broad scatter in abundance was registered for the main phyla, i.e., *Bacteroidota*, *Firmicutes* (*Bacillota*), *Proteobacterota* and *Actinobacterota*. The non-classified sequences comprised <3% at the phylum level and about one-third at the genera level (2287 of 7378 detected sequences).

The relative abundances for the 120 most common bacterial genera (detectable in ≥10% of total sample numbers) were assessed at different time periods of HSCT. As expected, most of the intestinal bacterial genera belonged to *Firmicutes* (*Bacillota*), a large phylum containing a number of anaerobic species.

Using the publicly available SILVA database of 16S rRNA sequences, we found a significant time-dependent reduction in bacterial alpha-biodiversity on days +20 to +60 after HSCT, as assessed both by Shannon and Simpson indexes (Figure 1B,C). We also performed a β diversity analysis, but we did not find significant variations between the samples.

**Figure 2:**
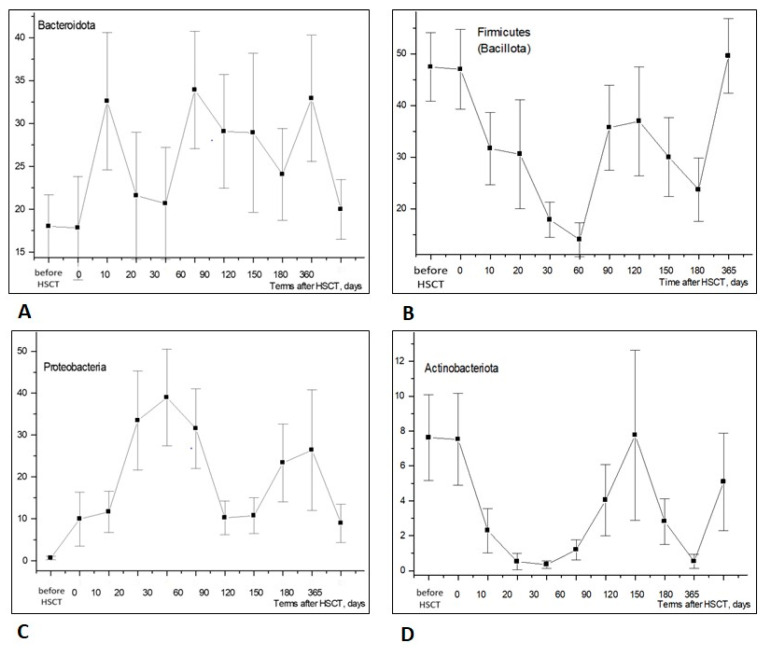
Relative contents of major bacterial phyla in stools of the HSCT patients—(**A**) *Bacteroidota*; (**B**) *Firmicutes* (*Bacillota*); (**C**) *Proteobacteria* (*Pseudomonadota*); (**D**) *Actinobacteria* (*Actinomycetota*)—at increasing terms after HSCT. Abscissa; terms after HSCT; days. Ordinate; relative contents of distinct bacterial phyla; percent of total bacterial mass (M ± m).

Figure 2 shows the time-dependent changes in different phyla of intestinal microbiota within early and later periods (up to 1 year) post-transplant. For example, *Bacteroidota*, a dominant phylum of microbiota, exhibited a broad scatter of results and did not show any significant dynamics during the first 90 days post-transplant (Figure 2A). Meanwhile, the relative contents of *Firmicutes (Bacillota)* and *Actinobacteria (Actinomycetota)* exhibited a sufficient drop to near-zero values within 2 months after HSCT (Figure 2B,D). Accordingly, the ratios of *Proteobacteria* (including clinically sound intestinal bacteria) were sufficiently increased at 20 to 60 days after HSCT (Figure 2C).

#### 3.1.2. Changes in Bacterial Genera

**Figure 3:**
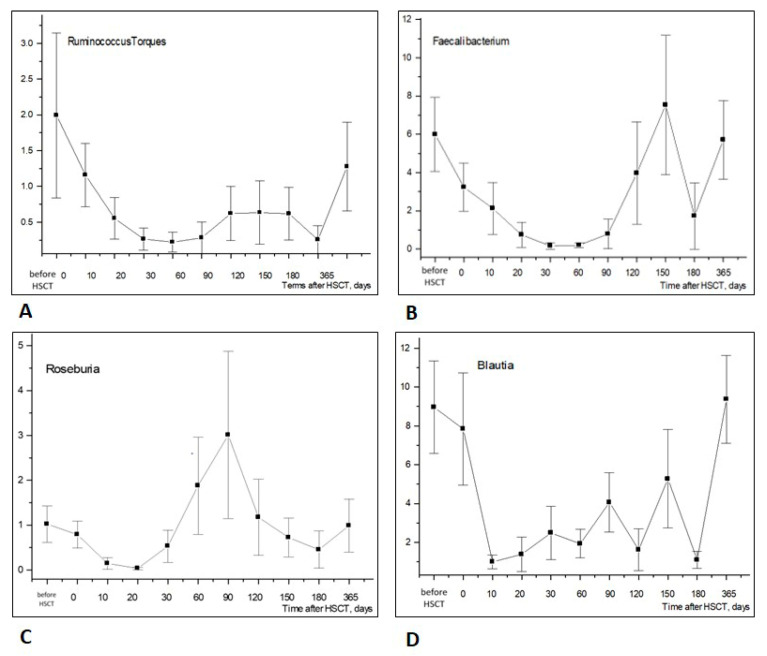
Time-dependent changes in Firmicutes (*Bacillota*) genera in stool samples after allogeneic HSCT: *Ruminococcus torques* (**A**), *Faecalibacterium* spp. (**B**), *Roseburia* (**C**), *Blautia* (**D**). Abscissa; terms after HSCT; days. Ordinate; relative contents of distinct bacterial phyla; percent of total bacterial mass (M ± m).

The time-dependent dynamics of bacterial phyla were associated with changes at the level of the appropriate genera. We revealed athesufficient exhaustion of some typical anaerobic bacteria from the *Firmicutes* (*Bacillota*) phylum at 10 to 20 days post-HSCT, followed by their recovery at later periods (3–4 months post-transplant), as shown in Figure 3.

**Figure 4:**
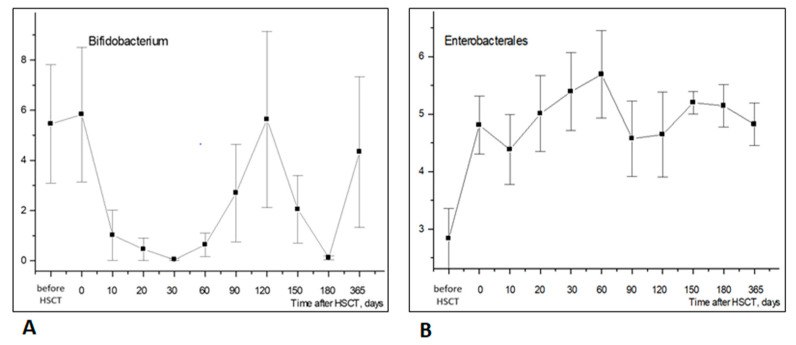
Longitudinal time course of *Bifidobacterium* (**A**) and *Enterobacterales* (**B**) abundance in stool samples after HSCT. Abscissa; terms after HSCT; days. Ordinate; relative contents of distinct bacterial phyla; percent of total bacterial mass (M ± m).

*Bifidobacteria* (phylum *Actinomycetota*) are also of clinical significance due to their known probiotic effects. Their relative contents in fecal microbiota were found to be decreased by 20–60 days post-transplant (Figure 4A).

The low incidence of the *Enterobacterales* order (*Proteobacteria* phylum) was observed before HSCT, followed by a rise within 10 to 30 days post-transplant (Figure 4B). Hence, *Enterobacterales* increased notably earlier than *Firmicutes (Bacillota)* and *Actinomycetota* in the post-transplant period.

### 3.2. K. pneumoniae Colonization and Antibiotic Resistance

**Figure 5:**
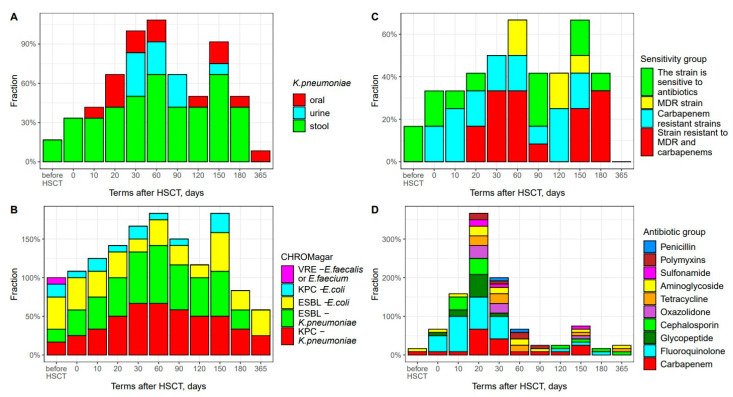
Frequency of antibiotic-resistant bacterial strain cases at different times after HSCT. (**A**) *K. pneumoniae* colonization sequence for different body sites. Abscissa; terms after HSCT TГCK; days. Ordinate; positive test results; percent of total group; (**B**) Time course of antibiotic-resistant fecal bacteria. Abscissa; terms after HSCT; days. Ordinate; positive test results; percent of total group. (**C**) Frequency of antibiotic-resistant fecal strains of *K. pneumoniae.* Abscissa; terms after HSCT; days. Ordinate; ratio of antibiotic sensitivity. (**D**) Escalation of anti-infectious therapy and groups of antibiotics applied. Abscissa; terms after HSCT; days. Ordinate; percentage of antibiotics used.

Gut colonization with *K. pneumoniae* was documented over the whole observation period, except day +365. Prior to allo-HSCT, two patients exhibited *Klebsiella* colonization according to the bacteriological data (Figure 5A). These strains were sensitive to the main groups of antibiotics (Figure 5C). Rectal swabs cultured on chromogenic media for the detection of resistant fecal bacteria showed positive results in six cases. Meanwhile, two patients exhibited combined positivity in CHROMagar ESBL and CHROMagar KPC tests for *K. pneumoniae;* ESBL-producing *E. coli* was revealed in five patients (Figure 5B).

At a later period, we observed a significant increase in cases with *K. pneumoniae* colonization, which was maximal on day +60 in 8 cases out of 12. The carbapenem resistance and multidrug resistance (MDR) of these bacterial isolates reached 67%, including two strains resistant to carbapenems, two MDR strains and four isolates with combined MDR and carbapenem resistance (Figure 5C). CHROMagar testing on day +60 showed positivity in 10 out of 12 patients. *K. pneumoniae* strains exhibited combined resistance in 89% of cases when using CHROMagar ESBL и CHROMagar KPC test media.

Upon extended *K. pneumoniae* colonization, we observed a decreased frequency of ESBL-producing *E. coli* (CHROMagarESBL medium) and carbapenem-resistant *E. coli* (CHROMagar KPC medium).

It should be noted that a second peak in intestinal *K. pneumoniae* colonization was registered by day +150 (8 out of 12 patients), with the resistance of bacterial isolates to carbapenems and other antibiotics (MDR phenotype) reaching 50% (Figure 5C). CHROMagar ESBL and KPC tests for *K. pneumoniae* were positive in 92% of cases (11 out of 12) patients.

One year after allo-HSCT, spontaneous *K. pneumoniae* decolonization may be documented due to the gradual recovery of both intestinal microbiota and patient’s immunity. At this time point, decreased amounts of *Proteobacteria* are revealed by both the NGS technique (Figure 2C) and bacteriological CHROMagar tests (Figure 5A,B).

All twelve HSCT patients were colonized with antibiotic-resistant *K. pneumoniae* at each time point. We traced the sequence of bacterial colonization for different sites (Figure 5A). Based on the bacteriology data, *K. pneumoniae* primarily colonized stool (since day 0), followed by throat samples (day +10). The urine specimens became infected at later terms, and were decolonized sometime earlier than intestinal sites.

**Figure 6:**
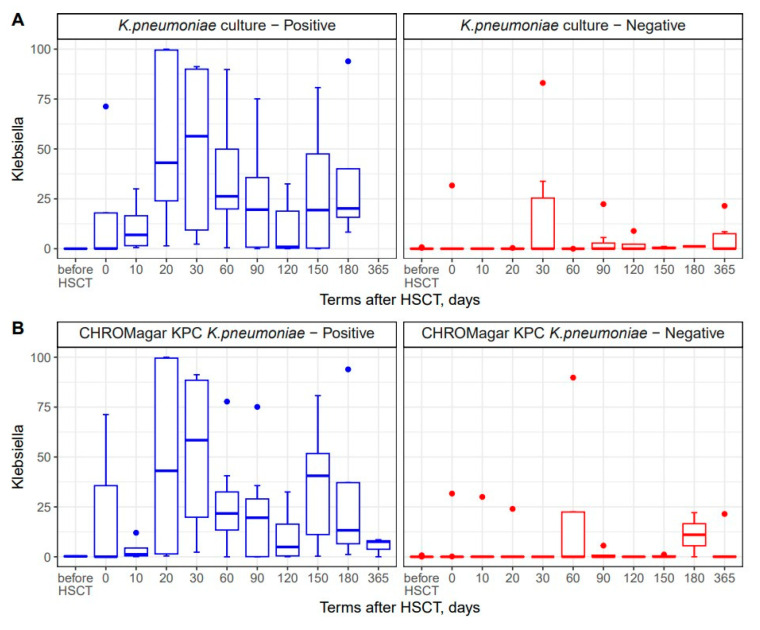
Comparisons between NGS technique and common bacteriology approaches to *Klebsiella* detection in stool samples (**A**) and a comparison between NGS-based detection and CHROMagar KPC culture of stool specimens (**B**). Blue bars show samples with positive results for bacterial cultures; red bars show specimens with negative *K. pneumoniae* cultures.

16S rRNA sequencing revealed the maximal abundance of *Proteobacterota* phylum by day +30 after HSCT (Figure 2C). *Klebsiella* spp. is a member of *Proteobacteria* phylum. We compared the bacteriology and NGS techniques in order to confirm these diagnostic approaches. In general, 16S rRNA sequencing allowed us to find *Klebsiella* spp. in the same specimens. However, some of the NGS-positive samples proved to be negative with routine bacterial cultures and/or CHROMagarKPC assays (Figure 6A,B). Furthermore, the relative contents of *Klebsiella* spp. determined by NGS in colonized (culture-positive) patients, were 30.4% (0.02–99.9) vs. 3.1% (0–83.1) in non-colonized samples. These results may suggest that the more sensitive detection of other *Klebsiella* species by DNA-based diagnostics, i.e., molecular biology methods (NGS and/or multiple PCR), may be required in order to identify *Klebsiella* spp. in complex biological samples.

### 3.3. Antibacterial Therapy and Infectious Complications

We used antibiotics within the first month post-HSCT and later on for the treatment of infectious complications. The peak escalation of the antibiotic treatment was registered on day +20 after HSCT, corresponding to the period of agranulocytosis and high risk of febrile neutropenia (FN). Cephalosporins, fluoroquinolones, glycopeptides and aminoglycosides were referred to as the first-line empirical therapy. Later on, carbapenems, oxazolidinones and polymixin were applied. The highest number of carbapenem-treated patients (8 out of 12) was registered on day +20 post-transplant (Figure 5D). In cases of *K. pneumoniae’s* isolation from blood or non-sterile body sites, the patients with FN or sepsis were administered targeted antiinfectiuos therapy based on antibiotic sensitivity tests.

FN was documented in 8 cases out of 12 (67%) on day +10 after HSCT. In five cases, FN was combined with intestinal *K. pneumoniae* colonization detected by bacteriology techniques. In seven out of eight patients, FN was accompanied by the positive findings of lactamase-producing Gram-negative bacteria, e.g., by means of cultures on CHROMagar ESBL (63%) and CHROMagar KPC (50%).

Sepsis was diagnosed in four patients, septic shock was diagnosed in two cases and bacteremia was documented in three patients. In three cases out of four, sepsis was accompanied by intestinal colonization, with *K. pneumoniae* being proven by CHROMagar ESBL and CHROMagar KPC bacteriological tests. Bacteremia was registered in three patients with sepsis, i.e., *K. pneumoniae* in two cases and *Pseudomonas* spp. in one case.

### 3.4. Temporal Shifts in Enterobacterales and Antimicrobial Resistance Genes

**Figure 7:**
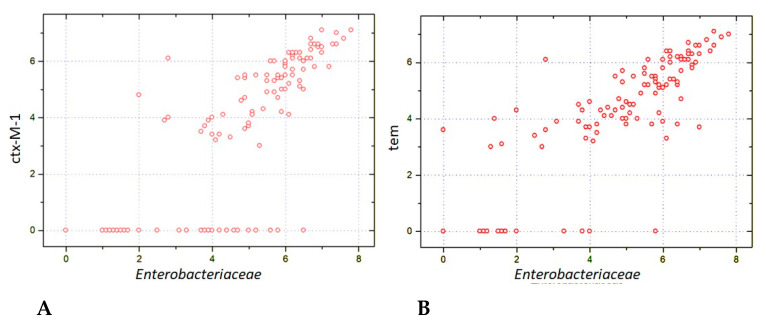
Correlations between relative contents of *Enterobacteriaceae* and carbapenemase genes: ctx-M-1 (**A**) and BLA_tem_ (**B**) in fecal samples from the patients after allogeneic HSCT. The respective correlation quotients are r = 0.816 (*n* = 119; *p* < 10^−8^; r = 0.829 (*n* = 119; *p* < 10^−8^).

The close relationship between relative amounts of *Enterobacteriaceae* and antimicrobial resistance genes (ARGs) was confirmed by means of multiplex PCR (Enteroflor and BakResista test systems). The results are expressed as the relative contents of bacterial and ARG genes in fecal samples. The scatter of points on Figure 7 shows that a sufficient fraction contained ARG-negative *Enterobacteriaceae* (zero values at the ordinate), whereas most samples showed detectable amounts of *bla*_CTX-M-1_ or *bla*_TEM_ resistance genes that were proportional to bacterial DNA contents (r > 0.8). Moreover, a moderate correlation was revealed between relative amounts of *Enterobacteriaceae* and the *bla*_SHV_ (r = 0.609; *n* = 119; *p* < 10^−8^) and with the *bla*_OXA-48_-like (r = 0.503; *n* = 119; *p* < 10^−8^). Thus, the post-transplant increase in *Enterobacteriaceae* is associated with the emergence of different lactamase genes also revealed by bacteriological techniques, e.g., CHROMagar systems.

**Figure 8:**
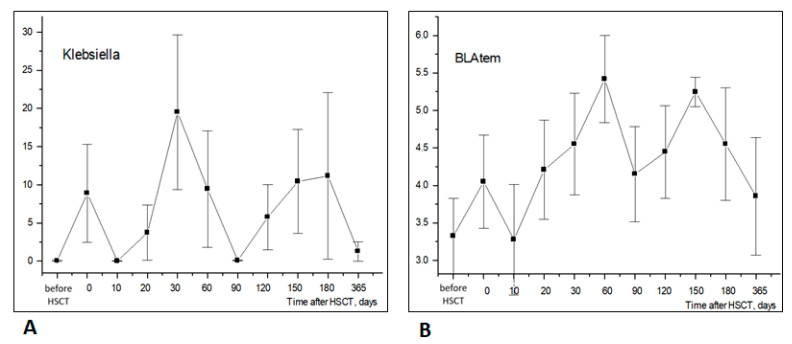
Similar time dynamics of *Klebsiella* ((**A**), NGS testing) and BLA_tem_ antibiotic resistance gene ((**B**), BakResista, multiplex PCR) in stool samples of HSCT patients (r = 0.515; *p* < 10^−9^; *n* = 120). Abscissa; terms after HSCT; days. Ordinate; relative contents of *Klebsiella* (**A**) or BLA_tem_ (**B**); percent of total bacterial mass (M ± m).

The relative abundance of *Klebsiella* belonging to *Enterobacterales* showed a sufficient increase compared to pretransplant levels by days +30 to +60, followed by a further increase on day +150 to day +180, before returning to near-basal levels by D + 360. Interestingly, the relative contents of *tem* ARG in stool microbiota shows a similar trend from the initial post-transplant period to 30–60 days after HSCT, thus probably reflecting the colonization of this site by ARG-containing strains of *Klebsiella* (Figure 8).

The time-dependent changes in *Klebsiella* (+) incidence were confirmed both by NGS and routine bacteriology cultures. In several samples, *Klebsiella* was revealed by NGS in culture-negative stool specimens, thus suggesting higher sensitivity of PCR diagnostics applied using the NGS platform.

The high abundance of *Klebsiella* was revealed by NGS in lactamase-positive specimens diagnosed by CHROMagar technique soon after HSCT and at later periods. These findings confirm the known incidence of antibiotic-resistant genes in *Klebsiella* strains in immunocompromised patients following HSCT.

Along with *Klebsiella*, some other common bacteria (except *Staphylococcus*) showed weak or moderate correlations with different lactamase ARGs (OXA-48, KPC, NDM, etc. (Table 2). Of them, *E. coli/Shigella*’s contents, as detected by NGS, showed similar correlations to *Klebsiella,* with KPC, BLA_ctx-M1_ and BLA_tem_ resistance genes being revealed by the BakResista multiplex PCR set. *Pseudomonas* ratios did weakly correlate with the NDM gene, whereas *Enterococcus* showed correlations with OXA-48, NDM, CTX-M-1 and BLA_shv_ genes.

*Streptococcus* and *Lactobacillus* did not exhibit any significant correlations with either of the ARGs from the BakResista PCR array.

As expected, the ratios of common *Firmicutes* (*Bacillota*), i.e., *Faecalibacterium*, *Ruminococcus*, showed negative correlations with *tem, ctx-M-1*, *shv* and *OXA-48*, thus confirming the general absence of these ARGs among sensitive anaerobic populations exhausted following HSCT procedure (Figure 3).

## 4. Discussion

To our knowledge, we are the first to trace the long-term shifts in intestinal bacteriome in patients after allo-HSCT and the pattern of its recovery, along with an analysis of colonization with antibiotic-resistant *K. pneumoniae*.

Our results confirm the early exhaustion of major *Bacillota* (*Firmicutes*) genera along with an increased ratio of *Enterobacterales* in the gut microbiota, associated with the higher incidence of some ARGs associated with *Enterobacteriales*. The bacteriome depletion seems to depend on the individual schedules of the administered antibiotics.

The initial period of 30–60 days after HSCT is considered critical for the evaluation of the suppression and subsequent recovery of hematopoiesis. The early exhaustion of most intestinal bacterial phyla after HSCT is observed during this period. Most authors attribute such disruption of intestinal microbiota to massive antibiotic treatment of infectious complications caused by profound immune deficiency following HSCT [14]. In our study, we observed an early exhaustion of anaerobic bacteria (phylum *Firmicutes*) even before the administration of antibiotics. The subsequent recovery of these bacterial populations was observed over the course of 2–4 months. Such time-dependent changes are in line with with the reported data on the depletion of certain anaerobic gut bacteria, e.g., *Ruminococcus, Faecalibacterium* spp., *Roseburia* and *Blautia* post-transplant, being accompanied by severe complications in HSCT patients [3]. The results are in accordance with the severe posttransplant dysbiosis at different mucosal sites post-HSCT, as shown elsewhere by routine bacteriology techniques [15].

The early exhaustion of *Firmicutes* (*Bacillota*) and *Actinobacteria* during conditioning might indicate that the bacteria also interact with chemotherpeutic drugs. It was shown that the presence of certain bacterial phyla decreases the clinical effect of chemotherapy [16]. The interactions between microbiome and chemotherapy agents might be diverse, including direct metabolism of conditioning agents [17], and selective cytotoxicity of drugs against certain bacterial phyla [18]. The intensity of the conditioning itself was previously shown to have a degree of impact on the microbiota injury after allo-HSCT [19,20]. Thus, the need for robust data on microbiome–chemotherapy interactions is clearly emerging. Furthermore, the mechanisms behind the selective expansion of antibiotic-resistant bacteria are largely unknown. It was demonstrated by whole-genome sequencing that the genetic characteristics of antibiotic-sensitive and antibiotic-resistant strains are quite different and involve genes regulating transport and binding proteins, energy metabolism, cellular processes and many other bacterial cell functions [21]. Early expansion of *Enterobacterales* in our patients after conditioning may suggest different interactions of these antibiotic-resistant strains with chemotherapy. The other explanation is the substitution of normal microbiota by nosocomial strains. However, the whole-genome sequencing was not carried out to determine the origin of antibiotic-resistant bacteria [22].

In general, the findings of our study are in accordance with the well-known decrease in bacterial diversity and dysbiosis following intensive cytostatic and anti-infectious therapy in HSCT cases. However, *Proteobacterota* phylum, e.g., *Klebsiella* and *E. coli/Shigella*, showed a trend toward early expansion, thus suggesting their selective outgrowth post-transplant. Special attention should be given to *Klebsiella* spp., a known intestinal pathogen of the *Enterobacteriaceae* family (*Proteobacterota* phylum), which is able to colonize the oral cavity and respiratory paths in immunocompromised patients [2,5]. In the present study, the late colonization of intestines with *Klebsiella* was confirmed by means of an NGS-based approach, showing the higher sensitivity of the NGS detection technique versus conventional cultures and CHROMagar KPC tests. Both early- and late-phase *Klebsiella* outgrowth have been associated with higher levels of detectable ARGs, especially those encoding β-lactamases. Time-dependent changes in gut microbiota were also monitored in the patients treated by HSCT and/or fecal microbiota transfer (FMT) using a qPCR-based panel of 47 different ARGs [23]. Long-term observations (up to 9 months) revealed a trend of reduced ARG incidence after FMT. However, the changes in distinct bacterial populations were not followed in this study.

In our group of patients, significant correlations were shown between the common *Enterobacterales* contents (*Klebsiella, E. coli* and *Pseudomonas*) and the presence of several ARGs (Table 2). Hence, a combined molecular biology assay using NGS-based 16S rRNA sequencing and multiplex PCR (Enteroflor and BakResista systems) may be an effective technique for the detection of *Klebsiella* strains resistant to β-lactam antibiotics in clinical settings, even in mixed real-world samples.

Among other factors, the gradual increase in ARG incidence in bacterial populations may be caused by the phage-mediated transfer of genetic material to the host bacteria, e.g., 16S rRNA gene typing in food and other biosamples has revealed numerous bacterial sequences integrated into phage DNA [24]. Similar resistance genes, i.e., *bla*_tem_, *bla*_ctx-m1_ and *bla*_shv_, were detected in the gut bacteria phages obtained from the hospital wastewaters [25].

In our study, relative contents of some β-lactamase genes, including *bla*_tem_, *bla*_ctx-m1_ and *KPC*, correlated with the relative abundance of *Klebsiella* and *E. coli*. Hence, a further aim of our work may be to address the ARG sequences integrated into the phage genomes of the human microbiome. This task may be resolved, e.g., by in-depth bioinformatic studies of the NGS data obtained with specialized primer panels, which covers a variety of bacteriophages hosted by the clinically significant pathogenic bacteria, especially, *Klebsiella, E. coli, Pseudomonas, Citrobacter*, etc.

There is a standard approach in clinical allo-HSCT to bacteriological confirmation of severe infections, along with emerging trend for assessing gut colonization with resistant bacteria, thus translating into higher risk of severe complications [25]. Several emerging clinical strategies are being developed to optimize antibiotic therapies in allo-HSCT recipients according to their colonization [26,27]. Thus, the observed sensitivity of ARG testing could also be evaluated as a clinical tool for decision making.

## 5. Conclusions

The significance of this work is that the changes in gut microbiome and ARGs were traced within 12 months post-transplant. The results obtained with NGS as well as the multiplex PCR of bacterial genes and ARGs in the stools of the patients after allo-HSCT confirmed previous findings on the early exhaustion of anaerobic bacteria after HSCT, probably due to the preventive antibacterial treatment, followed by their recovery at a later date. We have also revealed a partial replacement of the gut microbiota by the members of the *Enterobacteriaceae* family. Later infectious complications in these patients may result from the selection of antibiotic-resistant bacteria and their colonization, e.g., with *Klebsiella* spp. and *Enteroccoci*. The enterobacteria containing β-lactamase genes seem to accumulate at later terms in the gut microbiota, probably due to the suppressed immunity in post-transplant patients.

The present study has some limitations due to the small group of patients recruited for long-term observations. Different primary disorders and pre-treatment regimens seemed to produce greater scatter in the pre-transplant microbiome values and to prevent correlations with the individual clinical features of the patients.

Future studies should be performed in larger, more clinically homogenous groups to search for novel approaches to the efficient reconstitution of anaerobic microbiota in oncological patients after intensive cytostatic and anti-infectious therapy. Among such options, one may consider fecal microbiota transplantation, or replacement therapy with distinct microorganisms (*Bifidobacteria, Faecalibacterium* spp., *Roseburia*, *Blautia*, etc.), in order to restore normal microbiota post-transplant.

## Figures and Tables

**Table 1:** Clinical characteristics of HSCT patients.

Characteristic	Overall, N = 12 ^1^	Female, N = 4 ^1^	Male, N = 8 ^1^	*p*-Value ^2^
age	33 (30.25, 40)	30 (26.75, 34)	36 (31.75, 45)	0.2
HSCT donor type				0.2
Allogeneic related	3 (25.00%)	0 (0.00%)	3 (37.50%)	
Allogeneic unrelated	8 (66.67%)	3 (75.00%)	5 (62.50%)	
Haploidentical	1 (8.33%)	1 (25.00%)	0 (0.00%)	
Conditioning regimen				0.7
FluBe	1 (8.33%)	0 (0.00%)	1 (12.50%)	
FluBu10	2 (16.67%)	0 (0.00%)	2 (25.00%)	
FluBu12	9 (75.00%)	4 (100.00%)	5 (62.50%)	
Mucositis cases				0.7
0	2 (16.67%)	0 (0.00%)	2 (25.00%)	
1	2 (16.67%)	0 (0.00%)	2 (25.00%)	
2	2 (16.67%)	1 (25.00%)	1 (12.50%)	
3	5 (41.67%)	2 (50.00%)	3 (37.50%)	
4	1 (8.33%)	1 (25.00%)	0 (0.00%)	
Skin HVHD				0.7
0	7 (58.33%)	2 (50.00%)	5 (62.50%)	
2	1 (8.33%)	0 (0.00%)	1 (12.50%)	
3	4 (33.33%)	2 (50.00%)	2 (25.00%)	
Hepatic HVHD				0.2
0	9 (75.00%)	2 (50.00%)	7 (87.50%)	
1	1 (8.33%)	1 (25.00%)	0 (0.00%)	
2	2 (16.67%)	1 (25.00%)	1 (12.50%)	
Intestinal GVHD				>0.9
0	10 (83.33%)	3 (75.00%)	7 (87.50%)	
2	2 (16.67%)	1 (25.00%)	1 (12.50%)	
Chronic Skin GVHD				0.7
0	9 (75.00%)	3 (75.00%)	6 (75.00%)	
1	1 (8.33%)	1 (25.00%)	0 (0.00%)	
2	1 (8.33%)	0 (0.00%)	1 (12.50%)	
3	1 (8.33%)	0 (0.00%)	1 (12.50%)	
Chronic Hepatic GVHD				0.5
0	10 (83.33%)	4 (100.00%)	6 (75.00%)	
1	2 (16.67%)	0 (0.00%)	2 (25.00%)	
Chronic Oral GVHD				0.6
0	10 (83.33%)	3 (75.00%)	7 (87.50%)	
1	1 (8.33%)	1 (25.00%)	0 (0.00%)	
2	1 (8.33%)	0 (0.00%)	1 (12.50%)	
Sepsis				0.067
0	8 (66.67%)	1 (25.00%)	7 (87.50%)	
1	4 (33.33%)	3 (75.00%)	1 (12.50%)	
Septic shock				>0.9
0	10 (83.33%)	3 (75.00%)	7 (87.50%)	
1	2 (16.67%)	1 (25.00%)	1 (12.50%)	
Bacteremia				0.2
0	9 (75.00%)	2 (50.00%)	7 (87.50%)	
1	3 (25.00%)	2 (50.00%)	1 (12.50%)	

Notes: ^1^ Median (IQR); *n* (%), ^2^ Wilcoxon rank sum test; Fisher’s exact test. The following conditioning regimens were used: FluBe, Fludarabine phosphate and bendamustine; FluBu10, Fludarabine phosphate and Busulfan 10 mg/kg; FluBu12, Fludarabine phosphate and Busulfan 12 mg/kg.

**Table 2:** Intercorrelations between relative contents of common bacterial genera and detectable ARGs in stool samples of HSCT patients (*n* = 120).

Bacterial Genera	Antibiotic Resistance Genes (Multiplex qPCR )					
(16S rRNA Gene, NGS Assay)						
	OXA-48-like	KPC	NDM	CTX-M-1	TEM	SHV
*E. coli*/*Shigella*	r = 0.154	r = −0.305	r = 0.146	r = 0.325	r = 0.286	r = 0.024
	*p* = 0.05	*p* = 3.5 *×* 10^−4^	*p* = 0.05	*p* = 1.5 *×* 10^−4^	*p* = 8 *×* 10^−4^	*p* = 0.40
*Klebsiella*	r = 0.444	r = 0.249	r = 0.176	r = 0.468	r = 0.515	r = 0.680
	*p* = 2 *×* 10^−7^	*p* = 0.003	*p* = 0.03	*p* = 3.5 *×* 10^−8^	*p* = 9 *×* 10^−10^	*p* = 6 *×* 10^−18^
*Pseudomonas*	r = −0.061	r = −0.043	r = 0.268	r = 0.023	r = −0.064	r = 0.024
	*p* = 0.25	*p* = 0.32	*p* = 0.002	*p* = 0.40	*p* = 0.24	*p* = 0.40
*Ruminococcus*	r = −0.378	r = −0.149	r = −0.210	r = −0.356	r = −0.238	r = −0.410
	*p* = 1 *×* 10^−5^	*p* = 0.05	*p* = 0.01	*p* = 5 *×* 10^−5^	*p* = 0.004	*p* = 2 *×* 10^−6^
*Faecalibacter*	r = −0.284	r = −0.140	r = −0.183	r = −0.348	r = −0.257	r = −0.367
	*p* = 0.001	*p* = 0.06	*p* = 0.02	*p* = 5 *×* 10^−5^	*p* = 0.002	*p* = 2 *×* 10^−5^

## Data Availability

The data presented in this study are available on request from the corresponding author.

## Supplementary Materials

The following supporting information can be downloaded at: https://www.mdpi.com/article/10.3390/biomedicines12071566/s1, Figure S1: Rarefaction curves of NGS reads for the total group of patients. Sample size (X) is plotted against ASV (amplicon sequence variants, Y).
