# Supplementary material for: Time-Dependent Shifts in Intestinal Bacteriome, Klebsiella Colonization and Incidence of Antibiotic-Resistance Genes after Allogeneic Hematopoietic Stem Cell Transplantation
Source: Biomedicines. 2024 Jul 15;12(7):1566. doi: 10.3390/biomedicines12071566 (PMC11274722; doi:10.3390/biomedicines12071566)
Supplement: Supplementary file 1 [file biomedicines-12-01566-s001.zip › biomedicines-2981115-supplementary.pdf]

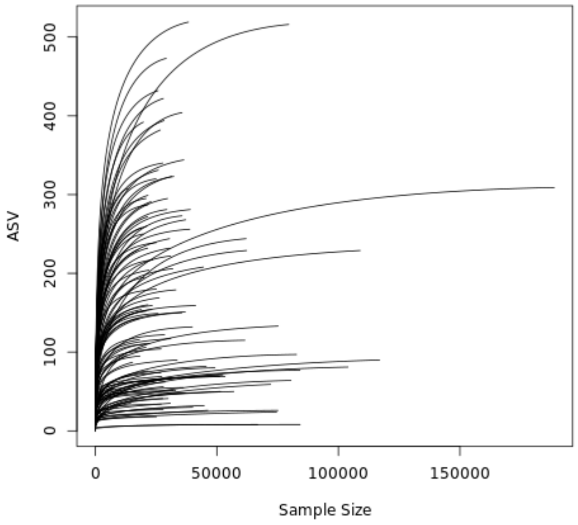

Figure S1. Rarefaction curves of NGS reads for the total group of patients. Sample size (X) is plotted against ASV (amplicon sequence variants, Y).
